# Whispering-gallery-mode resonators for detection and classification of free-flowing nanoparticles and cells through photoacoustic signatures

**DOI:** 10.1038/s41377-025-01978-9

**Published:** 2025-12-11

**Authors:** Jie Liao, Maxwell Adolphson, Hangyue Li, Dipayon Kumar Sikder, Chenyang Lu, Lan Yang

**Affiliations:** 1 Department of Electrical and Systems Engineering, Washington University, St. Louis, MO 63130 USA; 2 Department of Computer Science and Engineering, Washington University, St. Louis, MO 63130 USA; 3 AI for Health Institute, Washington University, St. Louis, MO 63130 USA

**Keywords:** Optical sensors, Optical techniques, Micro-optics

## Abstract

Micro and nanoscale particles have played crucial roles across diverse fields, from biomedical imaging and environmental processes to early disease diagnosis, influencing numerous scientific research and industrial applications. Their unique characteristics demand accurate detection, characterization, and identification. However, conventional spectroscopy and microscopy commonly used to characterize and identify tiny objects often involve bulky equipment and intricate, time-consuming sample preparation. Over the past two decades, optical micro-sensors have emerged as a promising sensor technology with their high sensitivity and compact configuration. However, their broad applicability is constrained by the requirement of surface binding for selective sensing and the difficulty in differentiating between various sensing targets, which limits their application in detecting targets in their native state or in complex biological samples. Developing label-free and immobilization-free sensing techniques that can directly detect target particles in complex solutions is crucial for overcoming the inherent limitations of current biosensors. In this study, we design and demonstrate an optofluidic, high throughput, ultra-sensitive optical microresonator sensor that can capture subtle acoustic signals, generated by tiny particles from the absorption of pulsed light energy, providing photoacoustic spectroscopy information for real-time, label-free detection and interrogation of particles and cells in their native solution environments across an extended sensing volume. Leveraging unique optical absorption of the targets, our technique can selectively detect and classify particles flowing through the sensor systems without the need for surface binding, even in a complex sample matrix, such as whole blood samples. We showcase the measurement of gold nanoparticles with diverse geometries and different species of red blood cells in the presence of other cellular elements and a wide variety of proteins. These particles are effectively identified and classified based on their photoacoustic fingerprint that captures particle shape, composition, molecule properties, and morphology features. This work opens up new avenues to achieve rapid, reliable, and high-throughput particle and cell identification in clinical and industrial applications, offering a valuable tool for understanding complex biological and environmental systems.

## Abstract (web summary)

This work introduces an optofluidic photoacoustic sensor enabling highly-sensitive, label-free, baseline-free detection of freely flowing particles and cells without surface binding or immobilization, offering robust, real-time analysis in complex media.

## Graphical abstract

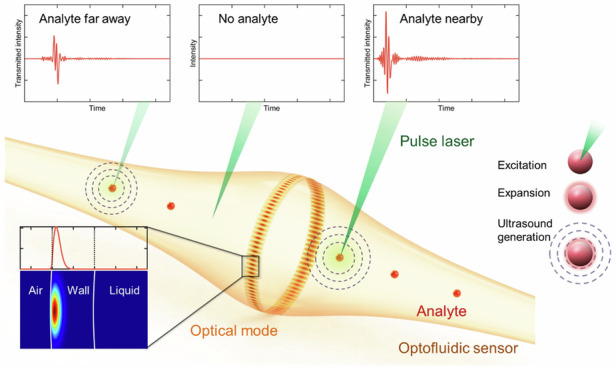

## Introduction

Particles in the micro and nanoscale are abundant in nature and play critical roles in diverse biological and environmental processes. Beyond their natural presence, these tiny particles are also present in a wide range of scientific research and industrial applications. Due to their small size, they possess unique physical, chemical, and biological characteristics markedly distinct from their bulk counterparts. Therefore, detecting, characterizing, and identifying these particles accurately and efficiently is crucial. In the field of medicine, for instance, nanoparticles are indispensable in targeted drug delivery systems, therapies, and diagnostics. They can interact with biological systems at the molecular level, enhancing treatment precision and efficacy^1,2^. In photothermal therapy, nanoparticles are used as agents that absorb light and convert it into heat, specifically targeting and destroying cancer cells^3,4^. In material science, nanomaterials exhibiting properties, such as increased strength, chemical reactivity, or electrical conductivity, open up new avenues for innovation in electronics, optics, and energy storage^5,6^. Given that the properties of particles depend on their composition, size, and shape, characterizing these particles becomes vital for understanding and leveraging the structure–property relationship^7^.

Spectroscopic techniques like Raman, photoacoustic (PA), and UV–Vis spectroscopy are widely used for characterizing particles and molecules. Raman spectroscopy, for instance, relies on the Raman shift, which results from inelastic scattering of photons interacting with molecular vibrations and other low-frequency modes in a sample^8^. The Raman spectrum can provide a direct molecular fingerprint that reflects the composition of materials. However, the detection efficiency in Raman spectroscopy can be low, posing a challenge, especially for nanoscale objects^9^. Raman scattering from water may also obscure the signals from target analytes in an aqueous solution, posing a challenge for conventional spectroscopic measurements. In recent years, various Artificial Intelligence (AI) techniques have been explored to improve the performance of conventional sensing^10^ and spectroscopy techniques^11^. PA spectroscopy combines optical excitation and acoustic detection, using short-pulsed lasers to excite acoustic signals generated by photon absorption and subsequent thermo-elastic expansion of targets. Analyzing the frequency domain features of photoacoustic signals provides insights into the shape, size, orientation of microstructures, and acoustic scattering properties, as well as various biophysical properties of the samples, making it a powerful tool for detailed characterization^12–16^. However, currently, this technique is mainly limited to nanoparticles with strong absorption, which are used as contrast agents for PA imaging^17^, due to the lack of highly sensitive sensors capable of detecting very weak acoustic waves generated by nanoparticles with low absorption.

**Fig. 1:**
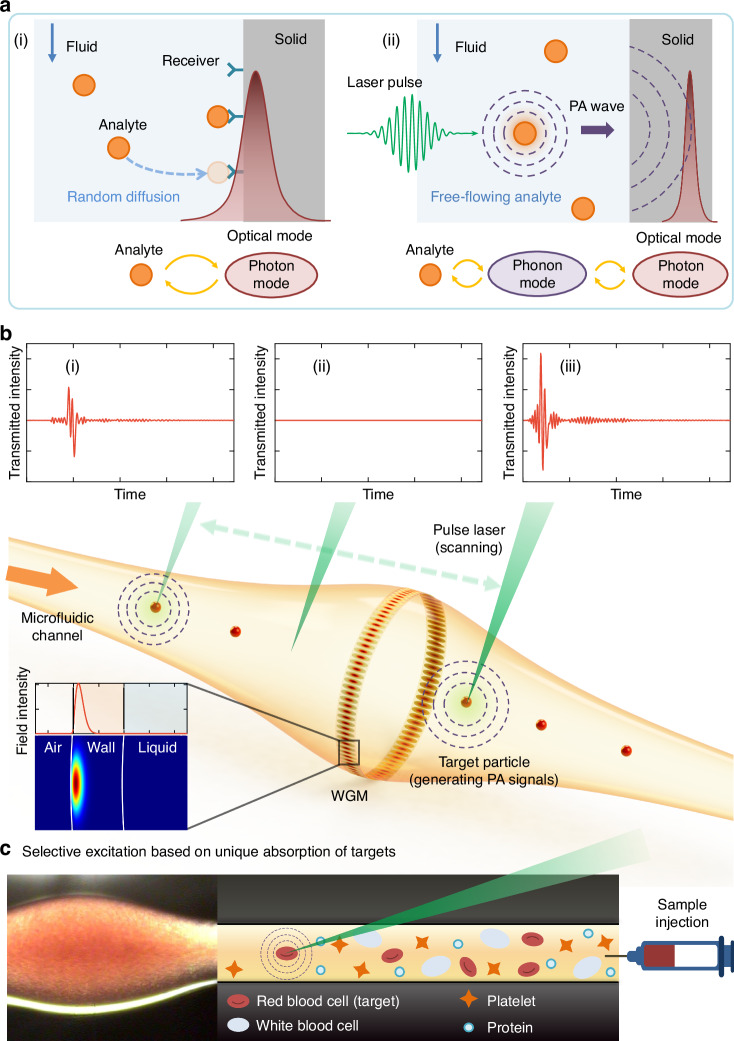
Label-free all-optical photoacoustic (PA) microresonator-based sensors. **a** Principle of long-range acoustic-assisted sensing. (i) Conventionally, particle detection through optical sensors is achieved by directly perturbing the photon mode (or hybrid mode by coupling with a plasmonic mode). These evanescent-field-based methods require the binding of particles on the sensor. Analytes flowing in the fluid cannot be detected effectively. (ii) In our approach, upon exposure to a pulse laser, analytes with absorption matching the laser wavelength generate PA waves propagating in the fluid medium. These PA waves can be efficiently captured by the high-*Q* optical mode confined within the dielectric structure. The acoustic phonons mediate a long-range interaction between light and the analyte particles flowing in the solution. **b** Schematic of the sensing platform using PA in an optofluidic microresonator. The red particles indicate analytes flowing inside the microfluidic channel. The green pulse laser excites the analytes at different locations, which can be captured by the whispering gallery mode (WGM) confined in the resonator. (i) PA signal from an analyte far away from WGMs. (ii) No PA signal is detected in the absence of the analyte. (iii) A stronger PA signal from an analyte close to WGMs. **c** Selective detection of red blood cells in a complex blood matrix. The left figure is a microscopic image when the microsensor is filled with whole blood. The right figure illustrates the specific excitation of PA signals in red blood cells in the presence of other cellular elements and proteins due to the unique absorption of hemoglobin in red blood cells at 532 nm

On the other hand, optical microsensors have emerged as a promising sensing technology in recent years, demonstrating great potential in small particle sensing due to their high sensitivity, rapid response, versatility, and affordability. The fundamental mechanism of detection involves analyzing alterations in optical signals as particles interact with light confined in the sensor. This provides information on various aspects of the target, such as its existence, concentration, and size^18–21^. Enhancing the light–matter interaction enables higher sensitivity; strategies including resonator-based sensing^22^, plasmonic enhancement^23^, and microlasers^24–26^ have been widely exploited, demonstrating the most demanding biosensing tasks, such as the detection of single virus particles and other nanoparticles^27–35^. However, in most cases, targets need to be in close proximity to or in direct contact with the micro-sensor’s surface for detection. For example, optical resonator sensors use evanescent fields to probe the surrounding media, and plasmonic-enhanced sensors rely on “hot spots”, posing a challenge in sample collection. Away from the surface, free-flowing particles in the sample solution remain undetected (Fig. 1a(i)). This means that only a small fraction of the target particles in the sample can be captured and analyzed, thereby limiting their detection efficiency and capacity for high-throughput sensing. Recently, photoacoustic effects have been utilized to enable the real-time measurement of the natural vibrations of mesoscopic particles^34^, as well as the detection and analysis of fluid samples^36^. In addition, measurements based on light intensity or phase, as well as resonance shift, provide little identifiable difference in the properties of different molecules or particles, as they are influenced by other factors such as the overlap of the light field with the analytes. While individual nanoparticles can be characterized by tracking their Brownian motion, the selectivity and specificity is limited^37^. To achieve selectivity and/or specificity in label-free sensing, the target molecules need to bind to receptors that are functionalized on the limited sensing surface. The need for surface binding and surface functionalization can introduce additional complexities and challenges, including surface fouling (non-specific binding), mass transport limitations (slow random diffusion rate, limiting the speed, sensitivity, and throughput), and surface regeneration (bound particles must be removed to reuse).

Moreover, to date, it is challenging for most optical microsensors, which are vulnerable to contamination, to obtain accurate measurements or detect specific targets in complex samples such as whole blood. The presence of numerous interfering substances, such as metabolites, electrolytes, and other biomolecules, can interact with the optical mode via evanescent fields, leading to false-positive or false-negative results and compromising the specificity and sensitivity of the detection system. To ensure accuracy, they often necessitate the implementation of intricate, multi-step sample purification protocols, including incubation and washing. Such procedural complexities pose significant challenges to their practical deployment in clinical and industrial environments. In addition, the complex nature of the sample matrix can cause signal attenuation, optical absorption and scattering, or background noise, further obscuring the target signal and making it difficult to obtain accurate quantitative measurements. Therefore, there is a strong need for label-free sensing technologies that can detect target particles directly in complex solutions, without the need for surface binding and purification. Such techniques would enable the detection and analysis of target particles in their native environment, potentially leading to higher throughput, simpler and faster detection, improved sensitivity, and greater overall efficiency in biosensing applications.

Here, we demonstrate an optofluidic, high-throughput, and label-free optical microresonator sensor with an extended detection volume, capable of real-time detection and interrogation of particles and cells in their native solution environments. The system is based on a high-quality (*Q*) bubble-shaped whispering gallery mode (WGM) microresonator, featuring a hollow core directly connected to a microfluidic channel that allows sample solutions to flow inside while being optically stimulated by a pulsed laser from the outside to generate acoustic waves through PA effects. The acoustic waves carrying the PA spectroscopic information of the samples can propagate through the solution and reach the WGM sensor, which could be positioned either close or far from the site where the acoustic waves are initially generated. Therefore, our approach enables fast and simple detection and measurement of micro and nanoscale objects across the microfluidic channel (Fig. 1a(ii)). In addition to exploiting photoacoustic effects for detection, it demonstrates, for the first time, the ability to detect and differentiate free-flowing particles that are beyond the reach of the evanescent field near the surface of an optical sensor, which is typically a limitation under conventional detection schemes. It is also worth noting that the resonator’s hollow core with a thick-wall design is ideally suited for integration with microfluidics. In this design, high-*Q* WGMs are fully protected within the thick wall, avoiding any direct overlap between the sample and the optical field of the resonant mode. This optical isolation from the sample ensures that both the quality factor and the signal-to-noise ratio (SNR) remain unaffected by the absorbing and scattering properties of the sample. Since the PA process produces acoustic signals originating from localized light absorption, the detection is free from optical background interference, remains robust against slow environmental changes, such as temperature fluctuations, and retains analyte-specific acoustic features even under varying environmental conditions. Our novel sensing platform not only ensure reliable and consistent results across multiple measurements but also enable direct target detection in complex sample matrices, such as whole blood, without the need for laborious sample purification. We leverage this novel sensing platform to facilitate high-throughput photoacoustic identification of particles and cells in liquid media using advanced machine learning approaches. This approach enables sensitive and specific detection of particles and cells within a small sample volume (~μL), making it an ideal solution for applications requiring rapid, accurate, and cost-effective analysis in complex biological matrices.

## Results

### Optofluidic PA WGM sensor

The working principle of our sensor system is illustrated in Fig. 1b, which includes a WGM microbubble resonator (MBR)^29,38–40^ built into a capillary to collect acoustic waves. The optical field of WGMs in the 780 nm band is confined within the silica wall. Solution samples containing particles flow through the fluidic channel connected to the MBR. When a particle flowing freely in the fluid is exposed to a pulsed laser at 532 nm, the energy absorbed by the particle causes a rapid thermoelastic expansion, resulting in the generation of an acoustic wave. These acoustic waves then propagate through the solution within the capillary to reach the WGM resonator. When an acoustic wave interacts with a WGM field, it alters the effective optical path length and consequently modulates the optical resonance. In this way, PA signals carrying information about the particles can be read out from the continuous wave laser coupled to the microresonator. The acoustic wave generation by sensing targets, e.g., nanoparticles, via the PA process, and the detection of these acoustic waves by WGMs are two crucial features of the sensing process.

The key advantages of this technique are label-free and immobilization-free detection, significantly enhanced detection volume, and direct target detection in complex biological matrices. Different from conventional optical WGM sensors, which detect refractive index changes near the sensing surface (see a comparison in Supplementary Fig. S1), our sensing system detects acoustic waves generated by particles in solution flowing freely inside a capillary structure, which significantly improves the sensing capability, throughput, and speed. Furthermore, unlike conventional methods that rely on surface binding via random diffusion, we can actively scan the pulse laser across the microfluid channel and search for target particles. As shown in Fig. 1b(i)–(iii), by scanning along the capillary, the particles far away from the MBR where the WGM resides can also be detected. The resulting PA signal exhibits a delay between the pulse excitation and its optical readout, due to the distance between the optical mode and the particles. We experimentally measured the PA signals from particles at various distances from the MBR and achieved an extended detection volume (Supplementary Fig. S3). In addition, by selecting the wavelength of the pulse laser that overlaps with the optical absorption of the particle of interest, the target particles can be selectively and effectively detected even in a complex fluidic medium. As shown in Fig. 1c, a whole blood sample is injected into the sensor. In such a complex medium, red blood cells can be selectively detected in the presence of other cellular elements as well as proteins, due to the unique absorption wavelength of hemoglobin in red blood cells.

The specificity and reliability are improved by making our sensor immune to dispersive (refractive index changes) and dissipative changes (optical loss) in the solution. We design the geometry of the microresonator such that the radial mode field is strongly confined within the thick wall of the MBR (Supplementary Fig. S2). With little penetration into the core with the solution sample, the optical WGMs are protected from either refractive index changes or potential absorption or scattering loss in the sample. Even if the core is filled with black dye solution, which has strong absorption, no apparent resonance shift or *Q*-factor deterioration is observed (Supplementary Fig. S2). Additionally, as we are detecting acoustic signals at high frequencies, the low-frequency noise (such as temperature drift and flow instability) is eliminated. In this way, the noise and false signal can be greatly mitigated and consequently improve reliability and robustness.

### Nanoparticle sensing

**Fig. 2:**
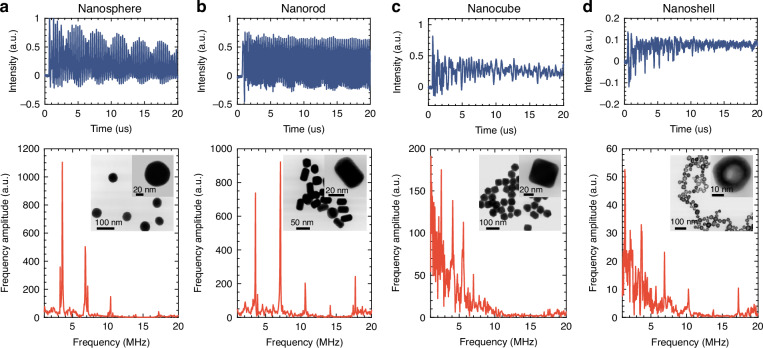
Temporal and spectral measurement of gold nanoparticles (AuNPs). **a–d** Time domain photoacoustic signals and corresponding frequency domain spectra of AuNP from four different geometries. In each panel showing the frequency-domain spectrum, the STEM image of the corresponding nanoparticles is displayed, with a zoomed-in inset highlighting an individual particle

To demonstrate the capability to detect and identify nanoparticles, four different geometries of gold nanoparticles (AuNPs) were tested in this study: nanospheres, nanorods, nanocubes, and nanoshells. Each solution was injected into the sensor via microfluidic channels. A pulse energy of ~40 nJ was used in the experiments to avoid photodamage, with a laser power of around 50 µW employed to excite WGMs. During pulsed excitation with deionized water in the core, no detectable PA signals or oscillations associated with optomechanical effects^41,42^ were observed. Figure 2 shows typical photoacoustic spectra for each of the four geometries and their corresponding frequency domain signals. Accompanying each time and frequency domain plot are scanning transmission electron microscope (STEM) images of each geometry. Note that the samples used for STEM images in the inset and for the PA sensing were handled differently. The STEM images show intentionally collected nanoparticle clusters, while the samples used for PA sensing were treated to prevent such aggregation. Care was taken to excite each type of AuNPs at the same position in order to standardize the collection of each signal. The PA signal variations among the four geometries of AuNPs are notably distinct. This indicates that different nanoparticle shapes, even when composed of the same material, lead to distinct spatial heat distributions that uniquely influence the photoacoustic signal generation^43^. This spectral information in the PA signal further provides a measure of the shape property of the particles. Once we obtain a library of PA fingerprints for various nanoparticles, a spectral matching algorithm can be implemented to find the closest match between the unknown spectrum and the library spectra. The reference spectrum with the highest similarity score is considered the most likely match for the unknown particle to achieve particle classification.

### Detection of cells and identification from different species

**Fig. 3:**
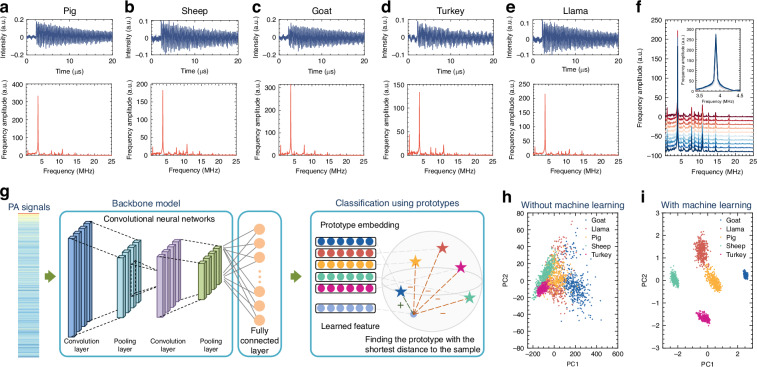
Photoacoustic signals of red blood cells and machine learning analysis. **a–e** Time domain photoacoustic signals with calculated frequency domain spectra of red blood cells from five different species of animals: pig, sheep, goat, turkey, and llama. The fast Fourier transform (FFT) spectra show very little difference between different kinds of cells. **f** Offset FFT spectra of 10 individual measurements of red blood cells from the same species (sheep). The vibration frequencies in the PA signal remain consistent across all measurements. Inset: Close-up of the peak near 3.9 MHz across different measurements without offset. **g** Schematic overview of the classification process and convolutional neural network (CNN) with prototype learning structure. FFT is performed on PA signals, followed by processing through the CNN. The CNN learns specific signal features during training, and then these features are used for classification by finding the nearest prototype embedding in the latent space. The model is eventually evaluated on the test set. Principal component analysis (PCA) is employed for feature visualization by simplifying the data into lower dimensions. **h** PCA visualization of samples without machine learning-based feature extraction. The resulting data points are intermingled, making them difficult to distinguish. **i** PCA visualization of samples using features extracted by the machine learning algorithm. The data points from each species form distinct, well-separated clusters. The results indicate that the machine learning-extracted features are effective in differentiating between the blood cells of various species compared to using raw signal features

Next, we explored the feasibility of biological particle detection and identification using photoacoustic fingerprinting. Here we tested five different species of washed red blood cells—pig, sheep, turkey, goat, and llama. These cell suspension samples were diluted to 1% by volume in a phosphate-buffered saline (PBS) solution, without any further purification, labeling, or incubation process. They were then injected into the sensing system and flown through the core of the capillary connected to the MBR. The temporal and frequency domain characterizations were obtained simultaneously, showing an SNR exceeding 30 dB. Figure 3a–e shows the time-domain and frequency-domain PA signals of the five different types of red blood cells. As shown in Fig. 3f, repeatability can be found in 10 measurements of red blood cells from the same species. In contrast with the results of AuNPs, the photoacoustic spectra of the 5 distinct cells look similar, with a major spectral peak near 4 MHz preserved across the species. Although there are some slight differences between different species, these differences may not be apparent to identify, and it could be challenging to distinguish the different species of cells directly. To tackle these challenges, we employed machine learning to analyze the differences at every single frequency component, which helped us identify them with high accuracy.

### Machine learning for cell classification from PA spectra

To analyze the unique PA signals and extract the features associated with different species of red blood cells, we carried out comprehensive measurements, compiling a substantial dataset of PA signals for each species. Given the notable similarities in the PA signals and spectra across various species, it is crucial to identify the subtle characteristics of each type of blood cell. As a powerful tool to extract meaningful information from complex datasets, machine-learning techniques were applied to learn from the PA signals by automatically identifying and extracting relevant features. The extracted features are more effective in distinguishing different types of red blood cells than the raw features.

To train the machine-learning-based feature extractor, we split the data into a training set (80%) and a testing dataset (20%). As shown in Fig. 3g, we first converted the input features from the time domain to the frequency domain using the fast Fourier transform (FFT). A one-dimensional convolutional neural network (CNN) is used to extract relationships among neighboring frequency components in the frequency domain of PA signals. The classification process begins by feeding the PA signals into the CNN, where the convolutional layer uses filters to extract features such as signal patterns. A pooling layer then reduces the data dimensionality, maintaining essential information as labeled in dashed red lines. The subsequent fully connected layer integrates these features, forming a comprehensive understanding to make predictions. To ensure robust predictions and mitigate the noise in signals, we further introduce prototype learning to enhance the similarity of the features extracted from the same cell species while reducing the chance of misclassification. The prediction depends on the sample’s distance to the prototype embeddings in the latent space of each cell category^44^. The model calculates the shortest distance from the learned feature to the prototype embeddings and classifies the sample as the cell category of its closest prototype embedding (see the “Methods” section and Supplementary Figs. S5–S7). Once trained, the machine learning model can analyze new, unseen PA signals and accurately classify them into one of the five species categories based on the features it has learned.

To illustrate the effectiveness of our machine learning approach in classifying PA signals, principal component analysis (PCA) was utilized to visualize the testing data with both raw features and the features extracted by the machine-learning model. PCA is a well-established technique for visualizing datasets by reducing their dimensionality. However, with only the raw features, the data of different species overlap considerably, making it challenging to differentiate the different species of red blood cells (Fig. 3h). In contrast, with the features extracted by our machine learning model, PCA reveals distinct decision boundaries between different categories, clearly separating the different red blood cell species, as shown in Fig. 3i. This demonstrates the significant improvement achieved by machine learning in classifying different types of blood cells, and the unique photoacoustic signatures can provide sufficient information to enable reliable classification.

These results demonstrate that our approach can not only detect the presence of free-flowing micro/nano particles in their natural environment but also obtain their photoacoustic properties. The frequency domain features of photoacoustic signals extracted by machine learning can be used to detect and classify different species of cells without the need for incubation, culturing, labeling, and imaging. We also characterized the PA signals on nanoparticle samples at varying concentrations. The PA signal intensity increased with higher nanoparticle concentrations, exhibiting a slope of 2.5 mV mL fM⁻¹ in the linear region. This makes the technique highly suitable for applications requiring precise detection of concentration gradients (Supplementary Fig. S4). With these findings, we propose a vision for rapid, PA detection and characterization that allows for particle/cell identification without the need for full spectroscopic and/or microscopic analysis, which typically relies on bulky equipment and complicated sample preparation.

### Detection of cells in whole blood

**Fig. 4:**
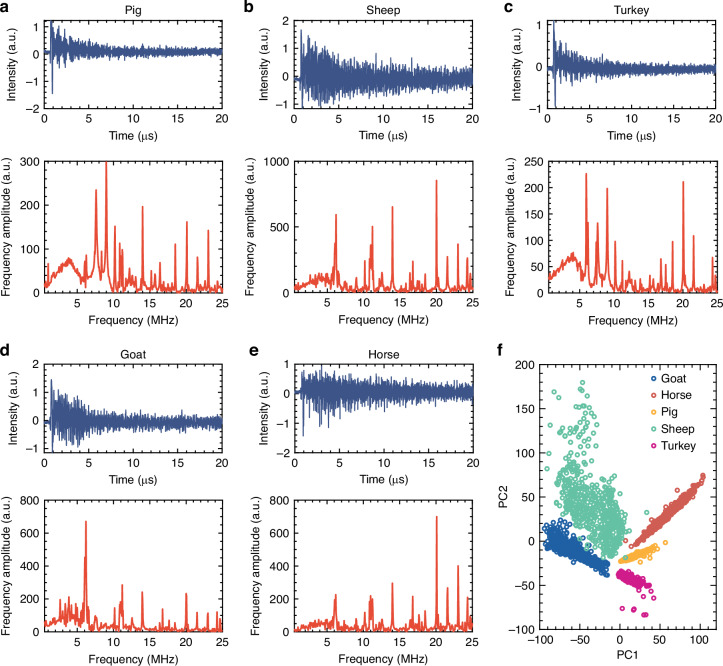
Photoacoustic fingerprinting of whole blood samples. **a–e** Time domain photoacoustic signals and corresponding frequency spectra of whole blood samples from five different species of animals: pig, sheep turkey, goat, and horse. **f** PCA visualization of samples using features extracted by the machine learning algorithm, indicating clear decision boundaries and clustering of each species in whole blood samples

The design of mode protection within solid walls of the MBR ensures that the optical resonances for photoacoustic detection operate effectively without being affected by the solution’s complexity. To explore the feasibility of sensing in complex matrix solutions, we tested whole blood samples of five different species: pig, sheep, turkey, goat, and horse by injecting the sample into the sensor and recording the corresponding PA signals. Whole blood is a complex biological fluid with colloidal properties, comprised of multiple components including water, cellular elements (including red blood cells, white blood cells, and platelets), various metabolites, dissolved electrolytes, a wide variety of proteins, and circulating hormones. The direct measurement of specific analytes in whole blood poses significant challenges for conventional sensor technologies. The presence of numerous biomolecules and cellular components can hinder the selective detection of target substances, leading to compromised accuracy and reliability in sensor performance. The blood samples tested in our experiments were diluted to 1% by volume, without any additional purification, labeling, or incubation process. Figure 4a–e shows the time-domain and frequency-domain PA signals of whole blood from five different species. The PA signal variations among them show characteristic differences that could be used as fingerprints for identification. The spectral features in the PA signal are related to the optical absorption, density, thermal, and mechanical properties in the whole blood samples. Due to the complex composition of the blood matrix, relatively large deviations appear in the PA signal across measurements (Supplementary Figs. S8b and S9). To classify the different whole blood samples, robust algorithms are required to identify common features within the same kind of whole blood, while ensuring that these features are distinct enough to differentiate between different types of whole blood. In whole blood, various factors, such as additional absorption, scattering, and the thermal properties of the fluid, may result in variations in the PA signal profile^45–47^. To address this, we trained separate machine learning models of the same architecture for whole blood and red blood cell samples on their respective datasets. The model trained on whole blood samples was specifically used for classification tasks involving whole blood. We employed a prototype learning model for the training process, where the model learns a uniform prototype representation for each species, ensuring that individual sample representations are aligned with the prototype. Figure 4f demonstrates the effectiveness of using PA signals obtained from whole blood samples for classification purposes. The clear and distinct decision boundaries between different categories highlight the ability to accurately differentiate between various sample types. Note that blood is a complex biological fluid containing various components that can interfere with conventional sensing techniques. However, the clear decision boundaries obtained from the PA signals suggest that this method is resilient to the inherent complexity of whole blood samples and allows us to accurately classify whole blood samples despite the inherent variability in the signal, making it a promising tool for blood-based analysis.

## Discussion

In summary, we have developed an optofluidic sensing platform that integrates highly sensitive optical modes and the photoacoustic effect to achieve rapid, label-free, and high-throughput measurements of particles. Unlike evanescent-field-based and SPR-based sensing platforms relying on surface binding for detection, our immobilization-free approach can detect free-flowing particles in fluid (away from the sensing surface), with excellent specificity achieved using PA spectroscopic signatures captured by a high-*Q* MBR. By confining optical modes within the wall of the MBR sensor, this technique enables long-range acoustic-mediated measurements without reliance on random diffusion and spatially decouples the optical mode from the sensing volume, providing great resistance to potential contaminations in the solution. The measurement does not require external fluidic channels and chambers, lengthy culturing, expensive reagents, or thermal cycling equipment, and remains effective under changes in the solution, such as variations in refractive index and absorption. This label-free and immobilization-free approach helps mitigate surface fouling, mass transport limitation, and surface regeneration challenges in conventional sensors. Furthermore, abundant information about the photoacoustic properties of particles can be obtained and implemented to classify the different morphologies of nanoparticles or different species of cells. Finally, our machine learning models achieve accurate classification, effectively distinguishing different types of particles and cells.

PA process exhibits intrinsic sensitivity to both the functional and molecular composition of the sample, leveraging the extensive optical absorption contrast present in biological systems. By choosing appropriate wavelengths to excite PA signals, we could selectively collect signals from specific objects. This unique capability allows for the non-invasive monitoring of a substantial number of red blood cells in their native physiological state when a 532 nm laser is used. Our approach circumvents the inherent complexities of whole blood, which often hinder conventional analytical techniques due to the potential for interference from the myriad of cellular and molecular components present in the blood matrix. Moreover, this technique minimizes the influence of external perturbations, such as temperature fluctuation and refractive index variations, to ensure an accurate assessment of the sensing targets through their PA signature. It is worth noting that the characteristics of the photoacoustic signals could be affected by various factors that influence the generation of the acoustic waves through transient thermoelastic effects induced by a pulsed laser. Photoacoustic generation in gold nanoparticle colloidal suspensions differs fundamentally from that in bulk solids or liquids. During laser exposure, the high thermal conductivity and nanoscale dimensions of AuNPs facilitate rapid heat transfer to the surrounding medium, which could affect the PA signals^47^. The photoacoustic generation is also affected by the laser pulse width, and the PA frequency of nanoparticles can be tuned from several MHz to hundreds of GHz by changing the pulse width^48^. High-frequency components may attenuate significantly in the far-field, and current microresonator bandwidth limitations may restrict detection within this range. Future improvements in sensor design could enable detection at higher frequencies. While we did not observe PA signals or optomechanical oscillations when the sensor was filled with water, mechanical resonances of the sensor may help enhance the detection of the PA waves. When the photoacoustic frequency approaches these mechanical resonances, the PA signals could be resonantly enhanced, potentially improving the detection sensitivity for weakly absorbing samples.

In principle, this technique can be applied to the detection and characterization of a wide range of targets in their natural states, offering versatility for sensing in both liquid and gaseous environments, such as cells in solution and viruses in air^49^. Both synthetic particles or biological cells can be measured with high throughput by choosing the proper wavelength of laser pulses or using a frequency comb^50^ in the PA process^51–53^. For instance, features in the PA spectra can be further used to study the status of red blood cells and differentiate diseased cells from healthy ones. This would enable rapid disease diagnosis, hemoglobin C disease, for example, or hemoglobin S-C disease, sickle cell anemia, and various types of thalassemia^54,55^. Highly automated measurement and data acquisition processes hold promise for clinical and industrial applications. When combined with large datasets, the AI-assisted sensing platform presented here could rapidly scan and identify cells in a patient sample and recommend an initial diagnosis in one step. It can also provide valuable particle information for nanomaterial characterization or environmental assessment without needing to wait for a culture or purification and random diffusion step. Such a smart sensing platform holds significant potential for advancing diagnostics, nanomaterial industries, and environmental monitoring.

## Materials and methods

### Sensor fabrication

MBRs were fabricated in line with a silica capillary (75 μm inner diameter and 125 μm outer diameter). First, we stripped away the polymer coating on the silica capillary using a butane torch and cleaned the silica window with isopropyl alcohol. Next, one end of the capillary was sealed with epoxy to allow the build-up of internal air pressure inside the capillary. The capillary was then placed onto a Vytran precision glass processing station and internally pressurized with air. The bare silica capillary was locally heated and inflated into a spherical geometry and form a microbubble resonator. The built-in microscope on the Vytran machine is used to monitor the fabrication process for quality control. By controlling the heating power and the applied pressure, the diameter as well as the wall thickness of MBRs can be controlled.

### Experimental setup

Figure 1b illustrates our MBR photoacoustic detection system. The built-in fluidic channels of the MBR are used to deliver the sample solution to the WGM resonator using a syringe pump. The syringe pump was set to withdraw the solution through the MBR at a rate of 10–50 μL min^−^¹. This flow rate ensures stable operation and reliable signal acquisition. No significant change in the PA signals was observed when the flow rate was varied.

WGMs were excited in the microresonator using a tapered 780HP optical fiber from Thorlabs. We used an external cavity diode laser centered around 780 nm connected to a function generator to scan the wavelength of the laser around 40 pm at a rate of 60 Hz. An optical attenuator and polarization controller were used to adjust the light intensity and polarization before coupling into the microresonator to optimize the WGM spectrum. The transmitted intensity from the microresonator was detected by a high-speed photodetector connected to an oscilloscope. Then we fixed the wavelength at an optical resonance by eliminating the scanning signal.

Photoacoustic excitation was achieved using a *Q*-switched 532 nm pulsed laser with a pulse duration of ~20 ns at a repetition rate of 60 Hz. This laser pathway is completely free space and focused on the MBR with an objective lens coupled to a CCD camera for optical alignment. This objective lens can also be scanned along the capillary axis, which means we are not limited to target detection just inside the MBR, but inside the capillary as well. The spot diameter (FWHM) is ~4 µm under the objective lens.

### Sample preparation

Four different geometries of AuNPs were used in this study, namely nanospheres, nanorods, nanocubes, and nanoshells. The AuNPs ranged in size from 10 to 50 nm and were suspended in a deionized water buffer with a citrate capping agent. The concentration of the colloidal AuNP ranged from 10^10^ nanoparticles mL^−^¹ (spheres, rods, and cubes) to 10^12^ nanoparticles mL^−^¹ (shells).

Five different species of whole blood as well as washed red blood cells were purchased from a commercial supplier (LAMPIRE Biological Laboratories). The whole blood was diluted to 1% in PBS solution and then delivered into the core of the sensor using a small syringe with a syringe pump. The suspensions of washed red blood cells were also diluted to 1% in PBS solution.

### Scanning transmission electron microscopy and imaging

For STEM images, 6 μL of different AuNP solutions were deposited on a pure carbon film having a mesh size of 400 and were then dried for 60 min at ambient temperature (~21 °C). The carbon surface adsorbed the AuNPs particles, and the water was evaporated. This carbon support film for STEM is very thin (15–25 nm) and highly transparent to electrons. The dried film is then mounted on the STEM sample holder for imaging.

### Machine learning of the PA signals

Details of the dataset and machine learning analysis can be found in Supplementary Session 5 (Fig. S5). For AI analysis for feature learning and particle classification, we employed a 1-dimensional Convolutional Neural Network as a feature extractor to learn useful information about the photoacoustic signal in the frequency domain. In the convolutional layer, multiple convolutional kernels stride along the vectors of the input, where each kernel can capture a unique local pattern in the spectrum, and subsequent pooling layers distill essential features. Then the feature maps, after the pooling process, are flattened into a vector and propagated into a fully connected layer with the activation function of Rectified Linear Unit (ReLU). Later, this learned feature was used for making predictions based on prototype learning. The detailed results of the machine learning models can be found in Supplementary Figs. S6 and S7.

### Prototype learning for robust prediction

A potential limitation of CNNs is that they tend to learn surface statistical regularities in the dataset rather than higher-level abstract concepts^56^. For highly sensitive optical sensors like WGM, trivial environment changes can be detected, and a well-trained CNN may misclassify with slight perturbations of the spectrum. Therefore, a robust machine learning model is required to assist in the detection and sensing.

In prototype learning^44^, prototype embeddings represent different classes in the latent space. The classification of a sample is simply implemented by finding the nearest prototype embedding using Euclidean distance in the latent space. The prototype embeddings are denoted as ${m}_{{yj}}$ where $y\in \,\{1,\,2,\,...,{C}\}$ represents the index of the classes and $j\in \{1,\,2,\,...,{K}\}$ represents the index of the prototype embeddings in each class. In our work, the number of prototype embeddings of each category is set to be *K* = 1, assuming that for each class there is only one representative embedding *m*_*y*_ in the latent space. These prototype embeddings, with a dimensionality equivalent to the feature space, can be initialized with trainable parameters so that they can be simultaneously updated with the model parameters in the training process.

For feedforward propagation, the prediction is determined by the distance between the sample $x$ and the prototype embedding *m*_*y*_ in the latent space instead of calculating the ${\rm{S}}{\rm{o}}{\rm{f}}{\rm{t}}{\rm{m}}{\rm{a}}{\rm{x}}$ function:

$$x\in {class}\mathop{\min }\limits_{i}{\rm{||}}{f\left(x,\,q\right)-m}_{y}{\rm{||}}$$

where *f*(*x*, *q*) denotes the output of the machine learning model (learned features) with trainable parameter *q*. Then, for the loss function, the probability of the prediction result is proportional to the negative distance $-{||}{f\left(x,{q}\right)-m}_{y}{||}$. Considering the non-negative of the probability and sum-to-one properties, this prediction can be written as

$$p(y|x)=\frac{{e}^{-||{f(x,q)-m}_{y}||}}{\sum _{k}{e}^{-||{f(x,q)-m}_{k}||}}$$

Therefore, a cross-entropy (CE) loss based on prototype learning can be described as

$$CE=-{\rm{l}}{\rm{o}}{\rm{g}}\,p(y|x)$$

Moreover, the robustness can be interpreted as the learned feature close to the prototype embedding in the latent space, which indicates that the model can neglect the noise and only preserve the key features of the class. Therefore, the loss function of prototype loss (PL) can be defined as minimizing the distance between the learned feature and the prototype embedding with the correct category:

$$PL={||{f(x,q)-m}_{y}||}^{2}$$

Eventually, the total loss can be defined as the sum of cross entropy loss (CE) and the prototype loss (PL):

$$loss=CE+\lambda \times {\rm{P}}{\rm{L}}$$

where *λ* is the hyperparameter that governs the influence of Prototype loss on feature extraction and decision boundary formation. As *λ* gets larger, the embedding of extracted features will get closer to the prototype. In the experiment, the hyperparameter *λ* is set to be 0.1.

## Supplementary information

Supplementary Information for: Whispering-Gallery-Mode Resonators for Detection and Classification of Free-Flowing Nanoparticles and Cells through Photoacoustic Signatures

## Data Availability

The data sets generated and/or analyzed during this study are not publicly available due to privacy and licensing restrictions. However, they can be made available from the corresponding author upon reasonable request.
