# Supplementary material for: Whispering-gallery-mode resonators for detection and classification of free-flowing nanoparticles and cells through photoacoustic signatures
Source: Light Sci Appl. 2025 Dec 11;14:397. doi: 10.1038/s41377-025-01978-9 (PMC12695887; doi:10.1038/s41377-025-01978-9)
Supplement: Supplementary file 1 — Supplementary Information for: Whispering-Gallery-Mode Resonators for Detection and Classification of Free-Flowing Nanoparticles and Cells through Photoacoustic Signatures [file 41377_2025_1978_MOESM1_ESM.pdf]

Supplementary Information for: Whispering-Gallery-Mode Resonators for Detection and Classification of Free-Flowing Nanoparticles and Cells through Photoacoustic Signatures

Jie Liao[1], Maxwell Adolphson[1], Hangyue Li[2], Dipayon Kumar Sikder[1], Chenyang Lu[1,2,3,4], Lan Yang[1,4]
[1]Department of Electrical and Systems Engineering, Washington University, St. Louis, MO 63130, USA
[2]Department of Computer Science and Engineering, Washington University, St. Louis, MO 63130, USA
[3]Department of Medicine, Washington University, St. Louis, MO 63130, USA
[4]AI for Health Institute, Washington University, St. Louis, MO 63130, USA
Corresponding author: yang@seas.wustl.edu

1. Sensing mechanism of long-range acoustic-mediated sensing

Conventional resonance shift sensing is a straightforward method to measure the target of interest by tracking induced changes in the resonance wavelength (or frequency) of an optical resonator. Fig. S1 (a) illustrates the resonance shift induced by target molecules captured on the resonator surface in many biosensing applications. This shift measurement allows for the extraction of quantitative and kinetic information about the binding of molecules. However, this method relies on random diffusion processes to bring particles to the sensing surface within the evanescent field, resulting in limited detection efficiency. Consequently, the detection is restricted to discrete binding events occurring at the sensing surface, providing only indirect or time-averaged information about the targets, and computation-intensive techniques are often required to extract more detailed information. Furthermore, the optical properties of the modes are directly modified by the analyte, making this method unsuitable for detecting large amounts of particles that exhibit scattering or absorption. Additionally, environmental factors can also induce resonance shifts, potentially affecting the sensing accuracy.

In contrast, acoustic-mediated sensing operates at a fixed wavelength and measures the transmission intensity changes induced by acoustic waves generated by the analyte, as shown in Fig. S1 (b). The acoustic waves generated by analytes propagate through the sample fluid and are detected by the optical mode, eliminating the reliance on random diffusion processes of targets to be captured on the sensor surface in conventional sensing. The intrinsic photoacoustic and mechanical properties of the analyte are encoded in the generated acoustic waves. In this method, the detection occurs through photon-phonon interaction, and the optical mode remains confined within the resonator without directly interacting with the analyte. As a result, acoustic-mediated sensing is not limited by the optical scattering and background absorption in the fluid. Moreover, since the photoacoustic process produces acoustic signals, the detection is free from optical background interference, providing a high signal-to-noise ratio and improved detection accuracy.

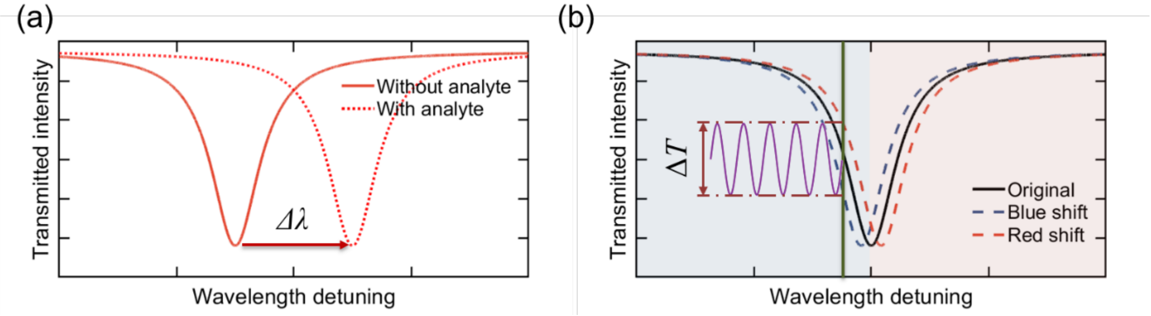

Figure S1: Comparison of sensing mechanisms in optical microresonators. (a) Conventional resonance shift sensing mechanism. The effective refractive index of the optical mode is modified by the binding of the analyte, resulting in changes of the resonance wavelength. (b) Acoustic-mediated sensing. The acoustic wave modulates resonance and induces optical transmission modulation ΔT at a specific wavelength.

2. Excellent stability for reliable measurement

Since the light field is fully confined within the thick wall of the microbubble resonator (MBR), direct interaction between whispering gallery modes (WGMs) and the sample solution is prevented. Consequently, when the core filling switches from deionized (DI) water to black dye,

there's a negligible change observed in the WGM spectra. This means that the Q-factor, along with the signal-to-noise ratio (SNR), and therefore the overall sensing performance, remains unaffected by the composition and refractive index of the sample media itself. This characteristic is critical for the sustained effectiveness of the sensors in various sensing applications over an extended period.

The sensitivity of WGM resonators can be enhanced by increasing their Q-factors. The smallest diameter that ensures material-loss-limited Q-factors depends on the resonator's geometry[1]. If the diameter becomes too small, radiation losses increase, which lowers the Q-factor. On the other hand, excessively large diameters offer diminishing returns in Q-factor enhancement while introducing practical drawbacks, such as the need for a larger sample volume during testing.

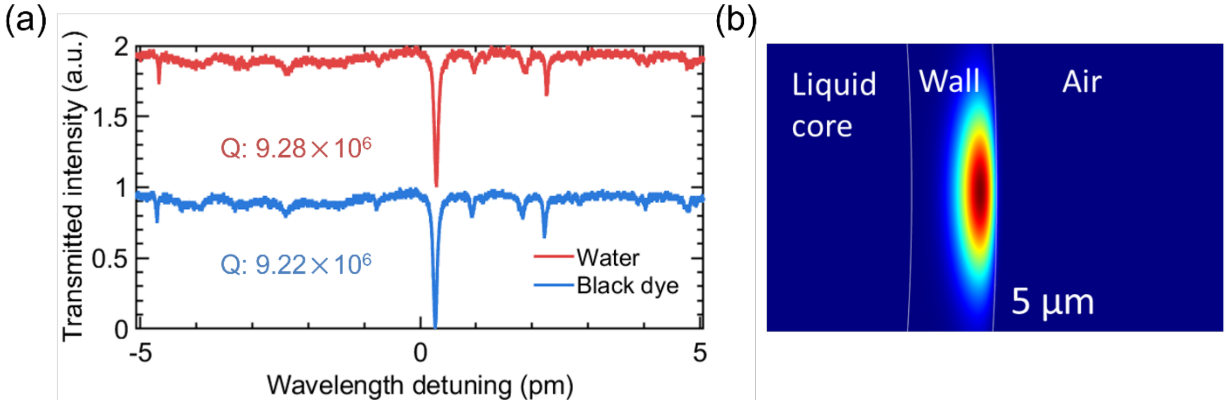

Figure S2: Thick wall for mode protection. (a) WGM spectra when the core of the sensor is filled with DI water and black dye, respectively. (b) COMSOL simulation of the field distribution of a WGM. When the wall is 5 µm thick, there is little overlap between the WGM and the liquid core in the sensor.

3. Extended sensing range

By adjusting the beam spot position of the pulsed laser, we achieve an extended sensing range, enabling the detection of particles even at distances far from the microresonator. Fig. S3 (a) illustrates the scanning of the pulse laser along the transparent capillary that serves as the microfluidic channel. To experimentally validate this expanded sensing range, we moved the pulsed laser's beam spot along the microfluidic channel while keeping the laser current constant. The distance between the WGM and the particles causes a delay between the pulse excitation and the optical readout. The measured delay time changes linearly with the distance of the pulse laser excitation location from the WGM sensor, as shown in Fig. S3 (b). This delay time can be used as an indicator that provides us with the position information of the particles. The signal amplitude, characterized as the peak-to-peak amplitude, drops at a larger distance. Even with the presence of loss, we can still obtain measurable signals at a very large distance of 6 mm. This capability, despite the inherent loss typically associated with the mechanical property of sample media and the capillary, demonstrates the extended reach of our sensing approach, making it particularly effective in scenarios where proximity to the target is a challenge.

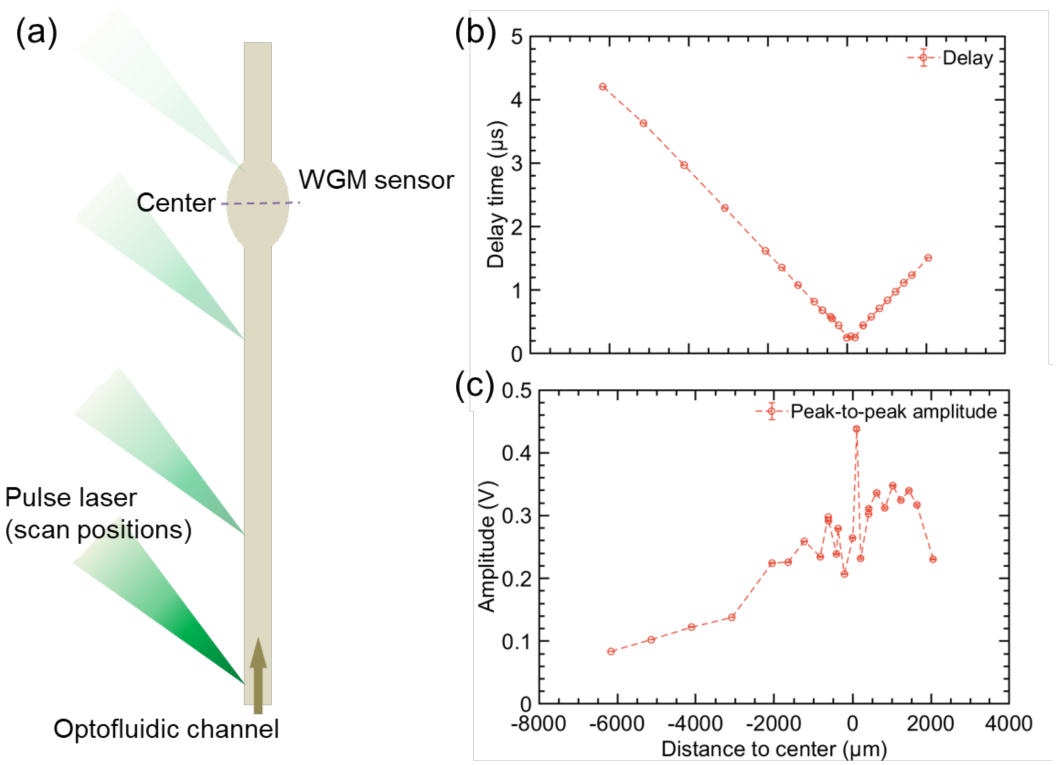

Figure S3: Extended sensing range. (a) Illustration of the scanning of the pulsed laser along the microfluidic channel, with WGMs being excited near the MBR equator while the sample solution flows through its core. (b) Time delays observed between the pulse trigger and the detected peak of the PA signals as the laser beam spot traverses along the channel. (c) Corresponding peak-to-peak amplitudes of the measured signals during the scanning.

4. PA signal amplitude as a function of nanoparticle concentration and input laser power

The photoacoustic response observed from the gold nanoparticles varies with the concentration of the nanoparticles in the solution and the input power delivered by the pulsed laser. Fig. S4 (a) shows that the photoacoustic signal amplitude increases with increasing concentrations of gold nanospheres. The dilutions were made from the original concentration of 3.28e10 nanoparticles mL^{-1} and the concentration of the diluted samples was confirmed with UV/Vis spectroscopy. The power of the pulsed laser was held constant at 37 µW and a repetition rate of 60 Hz. We see a near-linear relationship between the nanosphere concentration and the PA signal amplitude until about 3e10 nanoparticles mL^{-1} where saturation of the detector response is observed. Specifically, the slope in the linear region is 2.5 mV mL fM^{-1}, indicating the ability to detect and quantify concentration changes with high sensitivity. This makes the technique highly suitable for applications requiring precise detection of concentration gradients. In Fig. S4 (b) we show the dependence of the PA signal amplitude on the pulse laser power for a sample of gold nanospheres at a constant concentration of 3.28e10 nanoparticles mL^{-1}.

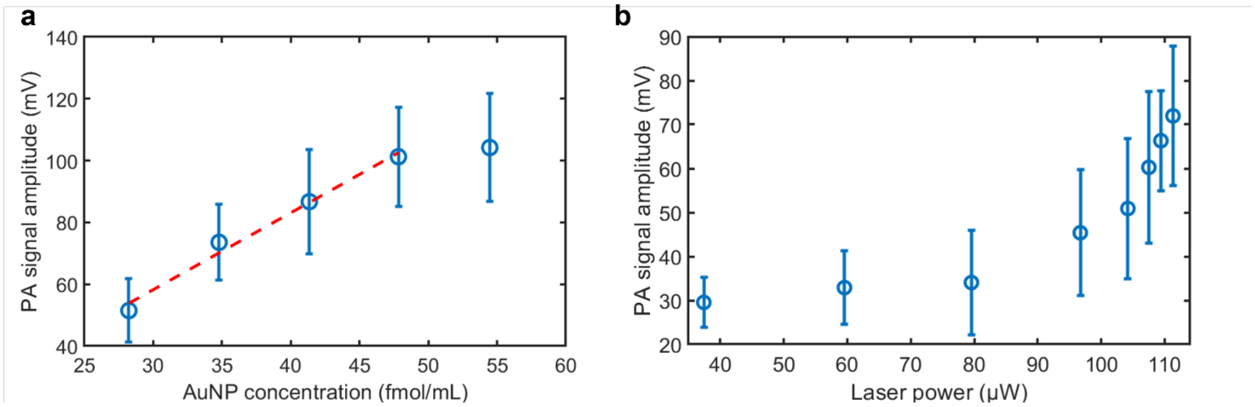

Figure S4: PA signal amplitude as a function of nanoparticle concentration and input laser power. (a) The PA signal response of gold nanospheres as the concentration is varied from 28.2 fM mL^{-1} (1.7e10 nanoparticles mL^{-1}) to 54.5 fM mL^{-1} (3.28e10 nanoparticles mL^{-1}). (b) The PA signal response of gold nanospheres at a concentration of 3.28e10 nanoparticles mL^{-1} under increasing power delivered by the pulsed laser.

5. AI analysis for feature learning and particle classification

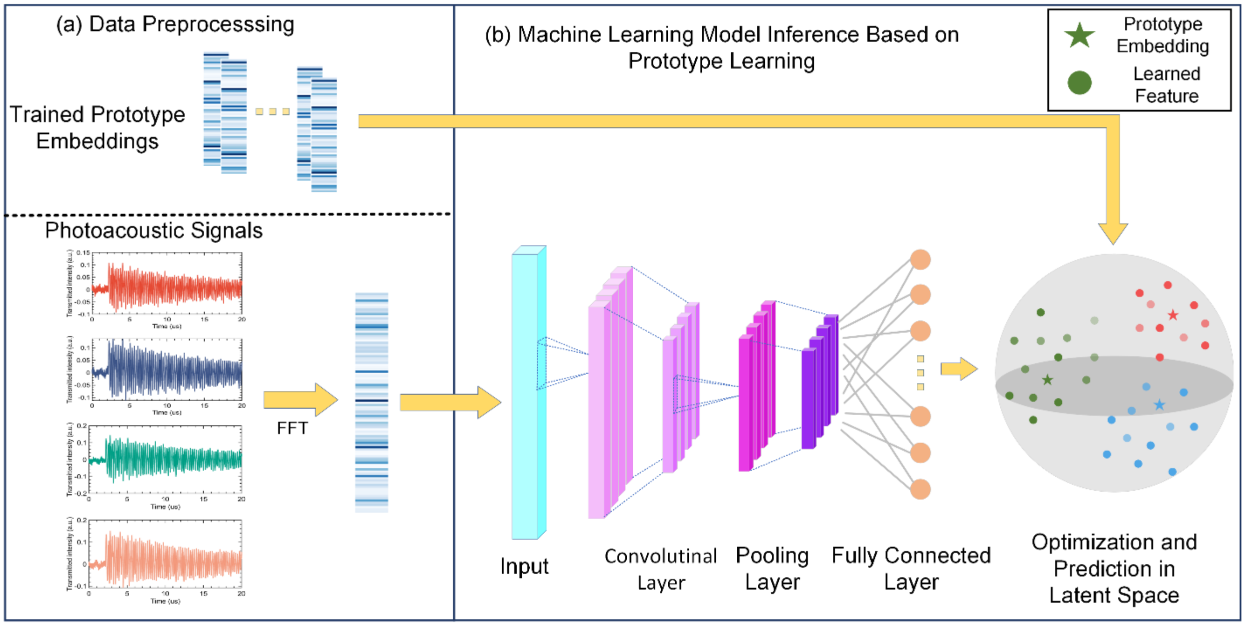

Figure S5: Machine learning pipeline for classifying PA signals. (a) The PA signals are preprocessed with the Fourier Transform as input data. The learned prototype embeddings of the different classes are used for inference. (b) The backbone model comprises a 2-layer CNN and a fully connected layer. The distance between the learned features of the sample and the prototype embeddings of each class is calculated in the latent space. Finally, the sample is classified as the class of its closest prototype embeddings.

5.1 Data description and preprocessing

The dataset collected from the experiments is used to train and test our machine learning model. The two distinct datasets from red blood cells and whole blood allow us to demonstrate the generality of the machine learning approach.

Red blood cells: This dataset contains five different blood cells for animals: goat, llama, pig, sheep, and turkey. For each species, 2000 photoacoustic spectra were collected such that the whole dataset contains 5000 different samples. For each sample, we use the Fourier Transform to transform the PA signals to the frequency domain, keeping the amplitude information as input features for the machine learning model. After preprocessing, the dimensionality of the input for machine learning is 10000. The dataset is separated into the training and testing sets with a ratio of 4:1, i.e., 8000 samples are used in training the model and 2000 samples are used to evaluate the model's performance.

Whole blood samples: This dataset is collected on the whole blood samples from five different animals: goat, horse, pig, sheep, and turkey. For each animal species, 2000 photoacoustic spectra are collected. Following the settings described above, the data were randomly split into training and testing sets with a ratio of 4:1. We trained the machine learning model to classify the blood samples from different animals.

Au nanoparticles: We can also implement machine learning to classify the particles based on their shapes for better accuracy. This dataset contains four different shapes of Au nanoparticles: nanocube, nanorod, nanosphere, and nanoshell. For each category, 1000 photoacoustic spectra are collected. We use a methodology similar to that used for the blood samples to process the Au nanoparticle data. We transform the PA signals to the frequency domain using the Fourier Transform while also keeping the amplitude information. The dataset is split into training and testing sets with a ratio of 4:1.

5.2 Model development

Convolutional neural network for photoacoustic spectra

Convolutional Neural Networks (CNNs)[2] is widely adopted for analyzing spectroscopy[3–5]. CNNs are specifically designed to learn spatial hierarchies of features from input data. This feature extraction capability is particularly beneficial for spectra, which often contain complex signatures that are difficult to manually engineer and identify.

For feature extraction, we employ a 1-dimensional CNN to extract useful information from the photoacoustic signal in the frequency domain. In the convolutional layer, four convolutional kernels stride along the vectors of the input, where each kernel captures a unique local pattern in the spectrum. The subsequent pooling layers distill essential features and reduce the computational cost. Then, the feature maps generated after the pooling process are flattened into a new vector and propagated into a fully connected layer with the activation function of Rectified Linear Unit (ReLU). After the activation function, the obtained vectors are the learned features to be used for classification in prototype learning.

Prototype learning for robust prediction

A potential limitation of CNNs is that they may learn surface statistical regularities in the dataset and cannot perform well with perturbations of input data, such as noise[6]. A highly sensitive optical

sensor (e.g., WGM) can detect small environmental changes. Consequently, small variations of the spectrum may lead to misclassifications by a well-trained CNN.

To enhance the robustness of the model, we implement prototype learning[7] (as described in Section Method). The prototype of the class is defined as the most representative embedding of the class in the latent space. In the training process, the model parameters and the prototype embeddings are simultaneously updated according to the gradient of the loss function.

With the learned embeddings of different classes, we classify new PA signals based on the distances from the learned feature of the PA signals to the prototypes of different classes in the latent space, as displayed in Fig. S5. The sample is classified as the class associated with the prototype that has the shortest distance to the sample's learned features.

Details of hyperparameters and implementation

The model is trained with 100 epochs and a learning rate of 0.001. Our training process also incorporates early stopping and a dropout rate of 0.3 to mitigate the effects of overfitting. The number of convolution layers is 2, and the stride of the convolution kernel and the stride of the pooling layer are set to 20. The number of the convolutional kernels is set to 4, i.e., the convolutional layer shares 4 different sets of parameters and can obtain 4 channels in the feature learning process. In the latent space, the dimensionality of the learned features, as well as the prototype embedding, is set to 64. For prototype learning, we train one prototype for each class. The model is programmed in Python 3.9.16 and Pytorch 1.8.0 and trained on RTX 3090 GPU, with a VRAM of 24 GB.

5.3 Model performance and evaluations

Evaluations on various metrics: To evaluate the performance of classifying the Au nanoparticles and the red blood cells, we made predictions on the testing data and measured these prediction outcomes with various metrics.

Accuracy: The proportion of correct predictions out of all predictions from the testing set.

Macro recall: The metric of recall is originally applied to binary classifications, which calculates the ratio of covered positive samples by the model: $\frac{True\_Positive}{True\_Positive+False\_negative}$. For classifications of multiple categories in our experiments, we use the macro recall defined as the mean value of recall in each class where the corresponding class is considered the positive label.

Macro precision: Similarly, the metric of precision is originally applied for the binary classification, which calculates the ratio of correctly predicted positive samples by the model: $\frac{True\_Positive}{True\_Positive+False\_Positive}$. For classifications of multiple categories in our experiments, we use the macro precision defined as the mean value of precision in each class where the corresponding class is considered the positive label.

Macro F1 score: The F1 score is the harmonic mean of precision and recall. The Macro F1 Score treats all classes equally, regardless of their frequency in the dataset.

The performance of each dataset is summarized in the following table:

Table 1: Overall Performance of machine learning on two tasks

Metrics	Accuracy	Macro Recall	Macro Precision	Macro F1
Au Nanoparticles	0.9961	0.9956	0.9971	0.9963
Red Blood Cells	0.9870	0.9863	0.9867	0.9864
Whole Blood Sample	0.9933	0.9934	0.9931	0.9932

The machine learning models achieve excellent predictive performance in classifying the PA signals in both datasets. The details of the result for each category are shown in the following confusion matrix:

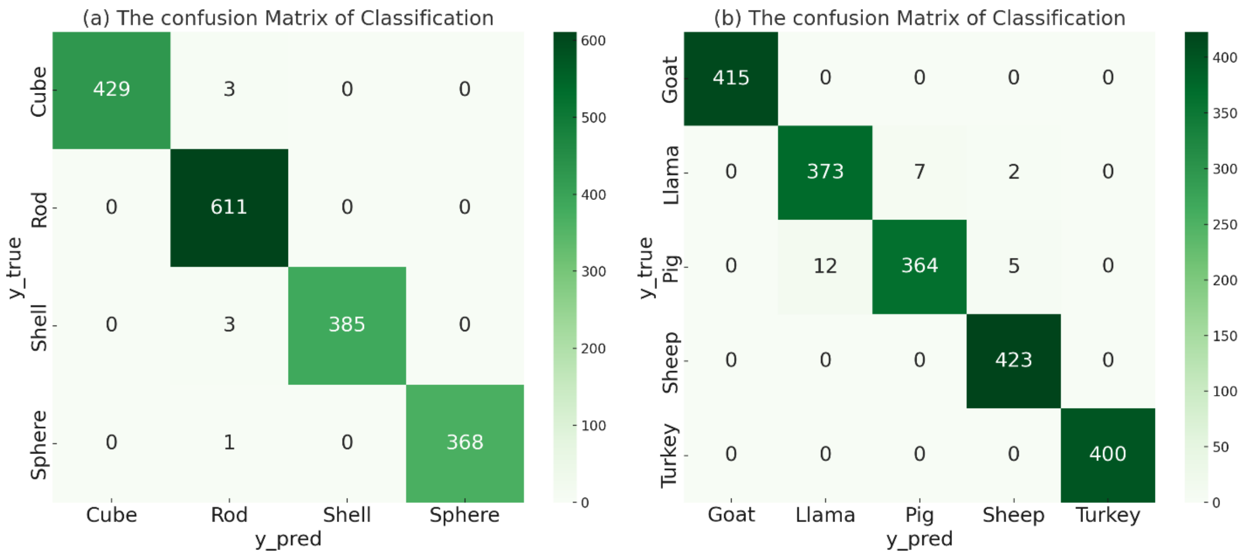

Figure S6: Confusion matrices of the machine learning models. (a) Result on Au nanoparticles. (b) Result on red blood cells.

Ablation study on prototype learning

We introduced prototype learning to make robust classifications. To assess the effectiveness of prototype learning, we compare the performance with and without prototype learning. The confusion matrices of the two approaches are displayed in Fig. S7. The original CNN struggles to distinguish the red blood cells of the pig and llama due to their significant similarities. In contrast, prototype learning enhances the model's ability to distinguish between llama and sheep.

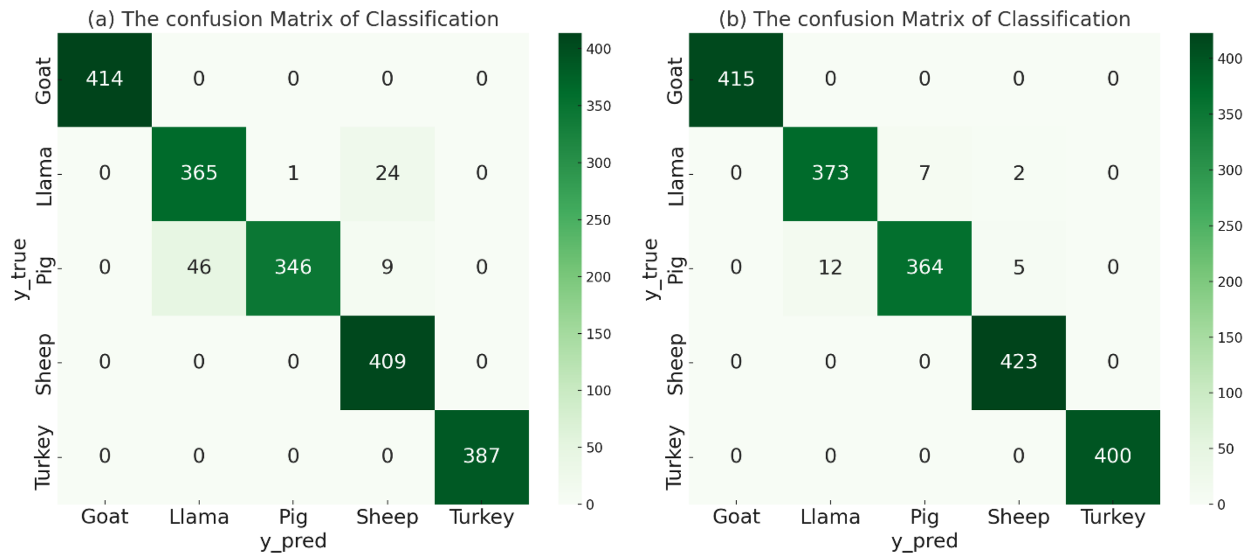

Figure S7: Confusion matrix on red blood cells. (a) Result without prototype learning. (b) Result with prototype learning.

6. Principal Component Analysis (PCA) without prototype learning

We performed PCA directly on the PA signals of red blood cells and whole blood samples, without the aid of AI. With only the raw features, the data of different species overlap considerably, making it challenging to differentiate the different species.

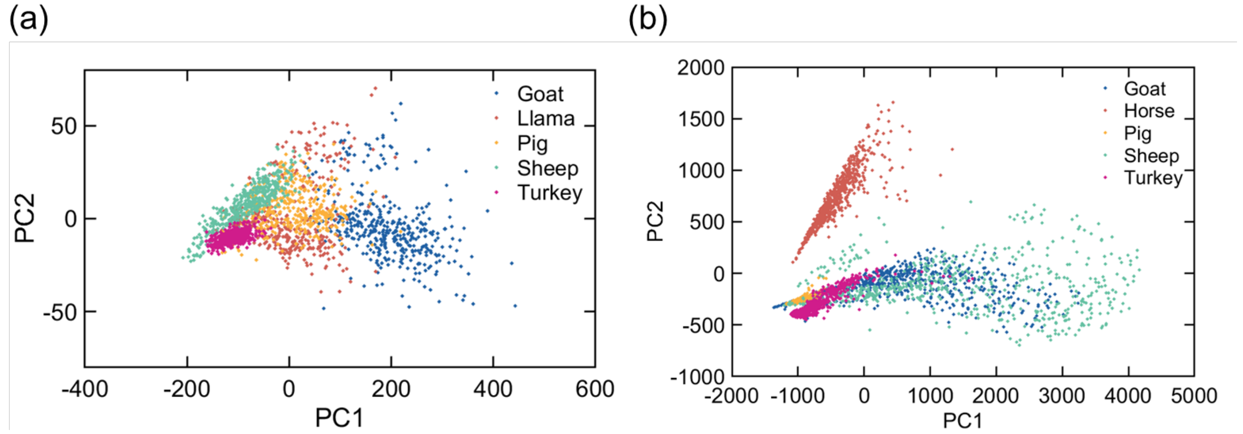

Figure S8: PCA without prototype learning. (a) PCA on input features from red blood cell signals. (b) PCA on input features from whole blood signals. Without prototype learning, the resulting data points are intermingled, making them difficult to distinguish.

7. Deviations in signals from whole blood samples

The PA signals obtained from whole blood samples exhibit larger deviations compared to those from red blood cell samples. These increased deviations can be attributed to the complex composition of whole blood. In addition to red blood cells, whole blood contains various other

components such as white blood cells, platelets, and plasma, each of which may contribute to the PA signal in different ways. The presence of these additional components introduces more variability in the PA signals, leading to larger fluctuations and a wider range of signal intensities.

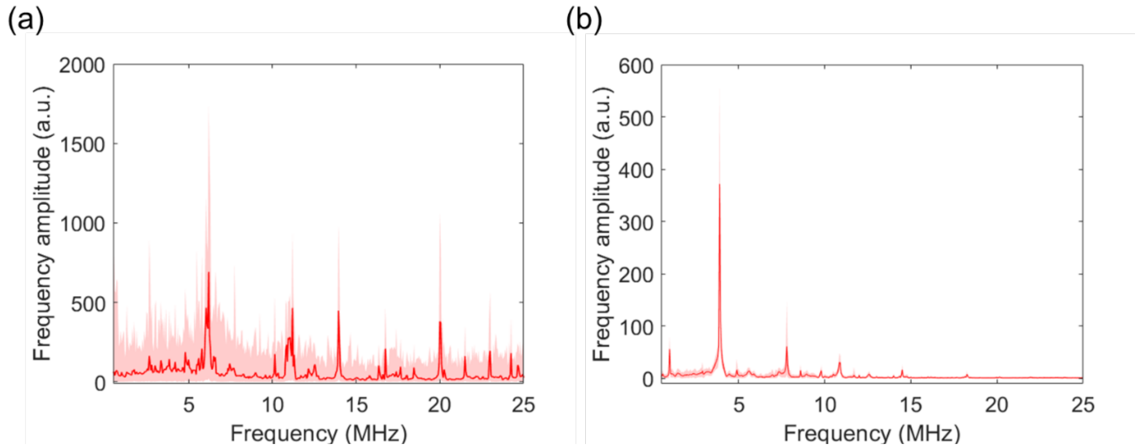

Figure S9: Deviations in the PA signals. PA signals from (a) whole blood and (b) red blood cell samples from goats. The red curve represents the average of 2000 frame signals. The pink-shaded area surrounding the red curve indicates the fluctuations in the PA signals, spanning from the minimum to the maximum values observed across the 2000 frames.

References:

1. Yang, Y., Ward, J. & Chormaic, S. N. Quasi-droplet microbubbles for high resolution sensing applications. *Opt Express* **22**, 6881-6898 (2014).
2. Lecun, Y., Bottou, L., Bengio, Y. & Haffner, P. Gradient-based learning applied to document recognition. *Proceedings of the IEEE* **86**, 2278–2324 (1998).
3. Kim, U. J. *et al.* Drug classification with a spectral barcode obtained with a smartphone Raman spectrometer. *Nature Communications* **14**, 5262 (2023).
4. Huang, L. *et al.* Rapid, label-free histopathological diagnosis of liver cancer based on Raman spectroscopy and deep learning. *Nature Communications* **14**, 48 (2023).
5. Seddiki, K. *et al.* Cumulative learning enables convolutional neural network representations for small mass spectrometry data classification. *Nature Communications* **11**, 5595 (2020).
6. Szegedy, C. *et al.* Intriguing properties of neural networks. in *2nd International Conference on Learning Representations, ICLR 2014* (2014).
7. Lundberg, S. M. & Lee, S. I. A unified approach to interpreting model predictions. *Advances in Neural Information Processing Systems* **30**, (2017).
